# Changes in oral microflora following 0.3% cetylpyridinium chloride‐containing mouth spray intervention in adult volunteers after professional oral care: Randomized clinical study

**DOI:** 10.1002/cre2.810

**Published:** 2023-12-02

**Authors:** Ai Fujimoto, Kana Fujii, Hirohisa Suido, Hisae Fukuike, Naoko Miyake, Hidenori Suzuki, Toru Eguchi, Haruko Tobata

**Affiliations:** ^1^ Research and Development, Sunstar Inc. Osaka Japan; ^2^ Department of Health and Nutrition, Faculty of Health Science Kyoto Koka Women's University Kyoto Japan; ^3^ Oral Health Promotion, Affiliated with the Sunstar Foundation Osaka Japan; ^4^ Sunstar Senri Dental Clinic, Affiliated with the Sunstar Foundation Osaka Japan

**Keywords:** 16S rRNA, Cetylpyridinium Chloride, Clinical Studies as Topic, Human Microbiome

## Abstract

**Objectives:**

This study explored the changes in bacterial flora composition and total bacterial count in the saliva and tongue coating, along with the change in the tongue coating index (TCI) following an intervention with 0.3% cetylpyridinium chloride (CPC) mouth spray after professional oral care.

**Materials and Methods:**

Fifty‐two adult volunteers aged 30–60 years were equally divided into CPC spray (*n* = 26) and control (*n* = 26) groups. All subjects underwent scaling and polishing. The CPC spray group was administered four puffs of CPC spray to the tongue dorsum four times a day for 3 weeks. The control group performed only routine daily oral care (brushing) and did not use any other spray. Bacteriological evaluation of saliva and tongue coating was performed using 16S ribosomal RNA gene sequencing and quantitative polymerase chain reaction. The tongue coating was evaluated to calculate the TCI. A per‐protocol analysis was conducted for 44 subjects (CPC spray group, *n* = 23; control group, *n* = 21).

**Results:**

At 1 and 3 weeks after CPC spray use, the flora of the saliva and tongue coating changed; the genus *Haemophilus* was dominant in the CPC spray group, whereas the genus *Saccharibacteria* was dominant in the control group. The sampling time differed among individual participants, which may have affected the bacterial counts. There was no significant intragroup change in TCI in either group.

**Conclusions:**

CPC spray affected the bacterial flora in the saliva and tongue coating, particularly with respect to an increase in the abundance of *Haemophilus*. However, CPC spray did not change the TCI. These results suggest that it may be optimal to combine CPC spray with a physical cleaning method such as using a tongue brush or scraper. Clinical Trial Registration: University Hospital Medical Information Network UMIN000041140.

## 1 INTRODUCTION

Preoperative oral management is recommended for patients undergoing cancer treatment and surgery under general anesthesia, as preoperative oral care has been shown to prevent the risk of postoperative infection (Ishimaru et al., 2018; Soutome et al., 2017). Whenever possible, before undergoing an operation, dental calculus and plaque are removed as professional oral care by a dentist or dental hygienist; however, in the period from receiving professional oral care to the operation, the patient must perform self‐care to maintain oral hygiene condition. Therefore, a simpler and more effective self‐care method is needed to promote patients' awareness and adherence to self‐care.

Mouthwashes containing chlorhexidine (CHX) gluconate or hydrochloride are generally used worldwide (Brookes et al., 2020); however, in Japan, the concentration of CHX permitted is limited because of the risk of anaphylactic shock (Okano, 1989; Urakawa et al., 2020). Therefore, cetylpyridinium chloride (CPC), a cationic surface‐active agent similar to CHX, is widely used as an oral care product (Lee et al., 2017; Shim et al., 2012). “GUM/Mouth & Throat Sterilization Spray” (Sunstar Inc.; hereafter referred to as CPC spray) is a self‐care product containing 0.3% CPC that is used to deliver CPC to the oral cavity at high concentration. Haraszthy and Sreenivasan (2017) performed a double‐blind, randomized study and reported that subjects who used a mouthwash containing 0.075% CPC for 4 weeks had lower gingival index (GI), plaque index, and bleeding index scores after 4weeks compared to their baseline scores. Similarly, Costa et al. (2013) conducted a 6‐month randomized parallel‐group comparison controlled study of 0.07% CPC mouth rinse in patients with moderate periodontitis; they reported lower mean plaque index and mean bleeding on marginal probing scores in the CPC group than in the placebo group, thus, demonstrating the clinical efficacy of CPC products. Teng et al. (2016) allocated patients to a 0.07% CPC mouth rinse group, a water rinse group, and subgingival and supragingival preventive treatment and tooth polishing were conducted in both groups. After 21 days of the intervention with CPC mouth rinse, the Mazza GI and bleeding on probing (BOP) were better than those in the water rinse group. Analysis of the α and β diversity of plaque bacteria further showed that accumulation of a new classification group was potentially blocked by CPC treatment, and the original diversity of plaque with healthy gingiva was maintained. Additionally, it was confirmed that the occupancy of 17 gingivitis‐related bacteria decreased in the CPC mouth rinse group (Teng et al., 2016). Thus, previous studies have examined the changes in clinical indices as well as the change in the microflora in the saliva and supragingival plaque induced by CPC mouth rinse (de Garcia‐Gargallo et al., 2017; Macedo Máximo et al., 2020; Teng et al., 2016). Because it is necessary to expel the mouth rinse, its use is limited. Conversely, since it is not necessary to expel the spray‐type product, CPC spray can be used easily without selecting a specific place of use, which may be accepted as a convenient self‐care tool. However, the bacteriological and clinical effects of CPC mouth spray have not yet been elucidated. For this initial study, we wanted to assess the potential changes occurring in healthy volunteers; however, in future studies, we plan to conduct this analysis for patients undergoing operations under general anesthesia. We aimed to compare changes in the oral microbiota with the use or nonuse of CPC spray among healthy volunteers.

Therefore, this study was performed as a randomized parallel‐group comparison study in adult volunteers to observe the changes in total bacterial count and bacterial flora composition in the saliva and tongue coating, along with the change in the tongue coating index (TCI) due to intervention with 0.3% CPC spray after professional oral care.

## 2 MATERIALS AND METHODS

### 2.1 Study design and subjects

This was a randomized, single‐blind, controlled, and parallel group comparative study performed at Sunstar Senri Dental Clinic, Affiliated with the Sunstar Foundation, from July to October 2020, and included adult volunteers aged 30–60 years. Recruitment of subjects was performed by 3H Medi Solution Inc., and the study information was published on their web media or email newsletter. The subjects meeting the following eligibility criteria were included: (1) provided written informed consent for participation; (2) male and female subjects aged 30–60 years at the time of acquisition of consent; and (3) having 24 or more natural teeth, including top, bottom, right, and left molars. Exclusion criteria were as follows: (1) taking any oral substances (e.g., antimicrobial drug, antibiotics) with potential impacts on the oral microflora; (2) using mouthwash, dental rinse, or interdental brush and dental floss; (3) conducting tongue cleaning daily; (4) smokers; (5) undergoing treatment for diabetes mellitus; (6) scheduled to undergo dentistry medical treatment during the study period; (7) pregnant or lactating women; (8) previously exhibited allergic symptoms to the test product or its formulation ingredient; (9) multiple caries (five or more C2); (10) severe periodontitis (marked inflammatory) (e.g., pus discharge, spontaneous bleeding, swelling, periodontal pocket, tooth mobility); and (11) any individual considered inappropriate for inclusion in the study by the subinvestigator.

### 2.2 Allocation/randomization of subjects

The subjects were stratified into groups according to values higher or lower than the median of the Shannon index, an index of α‐diversity of the microflora, in the saliva collected at the time of recruitment. Subjects in the two stratified groups were then randomly allocated to the two study groups using a random number table prepared by Microsoft Excel to ensure an equal distribution of baseline oral bacterial diversity in the two groups. To avoid bias due to differences in groups, blinding was performed by researchers not involved in the evaluation. Therefore, the dentist and dental hygienist in charge of the evaluation were blinded to the grouping of the participants.

The sample size required was calculated with reference to Teng et al. (2016). To have sufficient power to detect a difference in the Shannon index after 3 weeks between the CPC spray group and the control group, setting the intergroup difference in the mean Shannon index at 0.57, the standard deviation at 0.7 for the CPC mouth rinse group and 0.45 for the control group, the level of significance at 0.05, and the power at 80%, the number of subjects was calculated to be 38 using G*Power Version 3.1. Assuming a dropout rate due to lack of use of the CPC spray or loss to follow‐up to be within 20%, and to perform the substitute block randomization of a block size of 4, the number of subjects in each group was set at 24 (a multiple of 4). Therefore, a total of 48 subjects were set as the target number of cases in the study.

### 2.3 Study schedule and test product

The subjects visited the dental clinic at the time of recruitment (6 weeks before the start of the study), received an explanation about the details of the study, and gave consent. Saliva was collected for initial stratification according to the Shannon index, and laboratory tests in the oral cavity were performed. On Day 0 of the study, the tongue coating was evaluated to calculate TCI, and then samples of the saliva and tongue coating were collected. Scaling with an ultrasonic scaler (Solfy F; J. MORITA MFG. CORP.) and polishing with a polishing brush (Prophy brush ratch flat; Sakaki L&E Wise Co., Ltd.) and rubber cup (Prophy cup ratch 4 wave; EGUERU Inc.) were performed by the dental hygienist on all subjects as professional oral care. Subjects randomized to the CPC spray group were given the GUM/Mouth and Throat Sterilization Spray (Sunstar Inc.). On Day 1 after the start of the study, saliva and tongue‐coating samples were collected immediately after waking (see details of sampling methods below). After saliva and tongue coating sampling, subjects in the CPC spray group were administered four puffs (approximately 0.36 mL each) of the CPC spray to the tongue dorsum four times a day; three times after each meal of breakfast, lunch and dinner, and one time before sleeping. The subjects continued to perform their daily oral care routine. For example, if individuals in the CPC spray group typically brush their teeth after a meal or before sleeping, they use the CPC spray after brushing without rinsing their mouth with water immediately after use. The group without CPC spray was determined as the control group. After 1 week, samples of the saliva and tongue coating were collected at home from the subjects, similar to Day 1. After 3 weeks, saliva and tongue coating samples were collected at the dental clinic and the TCI was evaluated. Moreover, the subjects were requested to record the use of CPC spray and brushing of teeth, and the date, status of use of CPC spray, status of brushing of teeth, status of use of other oral care products such as mouthwash, status of taking the drug, and any unpleasant sensations and adverse events.

### 2.4 Clinical examinations

At the time of recruitment, the intraoral findings, including dental formula, probing pocket depth (PPD) (Glavind & Loe, 1967), BOP (Mombelli et al., 1987), GI (Löe & Silness, 1963), and plaque control record (PCR) (O'Leary et al., 1972), of all subjects were recorded at the time of recruitment. PPD was measured at all six sites of the teeth (mesiobuccal, buccal, distobuccal, mesiolingual, lingual center, and distolingual) using a probe (Periodontal Probe #5; YDM Corp.). For BOP, the bleeding that occurred after probing was evaluated according to the following four grades at all six sites of the teeth, similar to PPD: 0, no bleeding; 1, petechial bleeding; 2, splinter (zonal) bleeding; and 3, spontaneous bleeding. The GI was evaluated according to the following four grades at four sites (buccal, lingual, mesial, distal) of the target teeth (maxillary right first molar, lateral incisor, maxillary left first premolar, mandibular right first molar, mandibular left lateral incisor, mandibular left first premolar): 0, normal gingiva; 1, mild gingivitis (slight change in color, mild edema, no bleeding by probing); 2, moderate gingivitis (red, edema, sheen, bleeding by probing); and 3, severe gingivitis (marked red, edema, ulcer, spontaneous bleeding). Regarding PCR, all teeth were divided into four blocks, mesial, distal, buccal (labial), and lingual, and the dental plaque adhered to the tooth surface was checked. The number of blocks to which the dental plaque finally adhered was divided by the total number of blocks (four times the number of residual teeth) to calculate the individual PCR. Regarding the TCI, the tongue surface was divided into nine blocks, and the degree of adhesion of the tongue coating in each block was evaluated according to the following three grades: 0, no tongue coating observed; 1, thin tongue coating, possible to recognize the lingual papilla; 2, thick tongue coating, impossible to recognize the lingual papilla. The total score was divided by the maximum value (18) to calculate the individual TCI (Shimizu et al., 2007).

### 2.5 Sampling method

At the time of recruitment, Day 0, and Week 3, the subjects who underwent sampling in the morning stopped drinking and eating after breakfast, and those who underwent sampling in the afternoon stopped drinking and eating after lunch (drinking water was permitted). The sampling time was between 10:00 and 17:00 on Day 0 and at Week 3. The subjects stopped drinking water, gargling and smoking for 30 min before sampling. At Day 1 and Week 1, the subjects underwent sampling immediately after waking up. All saliva and tongue coating samples were collected using an Intraoral Flora DNA/RNA Sampling Kit (saliva: OMNIgene®/ORAL OM‐505; DNA Genotek; tongue: OMNIgene®/ORAL OMR‐120; DNA Genotek) and stored at 15–30°C (saliva) or 15–25°C (tongue). All collected samples were sent to Shizuoka Innovation Center (Shizuoka, Japan), Sunstar Inc.

### 2.6 Bacteriological evaluation

#### 2.6.1 Extraction of DNA

The collected saliva sample was mixed using a vortex mixer for more than 10 s and reacted in a water bath at 50°C for 1 h. Then, 200 µL of the sample was dispensed into an Eppendorf tube (DNA LoBind Tube; Eppendorf) and allowed to react in a heat block at 75°C for 15 min. Because precipitates were identified in the sample, the sample was centrifuged at 15,000 rpm and 4°C for 10 min to separate the supernatant and precipitate. Next, 180 µL of achromopeptidase (FUJIFILM Wako Pure Chemical) was added to the precipitate and reacted at 55°C for 10 min. The supernatant and precipitates were separately treated and adsorbed onto the same column of the Virus Spin Kit (QIAGEN). After lysis with AWI Cleaning Buffer, the supernatant and precipitates were cleaned with a single column and extracted with 100 µL of UltraPure^TM^ Distilled Water (Thermo Fisher Scientific).

The tongue coating sample was spiked with 1250 units of Ready‐Lyse Lysozyme Solution (Lucigen) prepared with 4 µL of TES buffer (1 mM EDTA, 10 mM Tris‐HCl [pH 7.5], and 100 mM NaCl), and incubated at 37°C overnight according to the recommendations of OMNIgene OMR‐120. The sample was then centrifuged at 15,000 rpm and 4°C for 10 min, and the supernatant and precipitates were treated in a similar manner as described above for the saliva.

The concentration of DNA extracted from the saliva/tongue coating samples was measured using SimpliNano (GE Healthcare); distilled water was used as the background.

#### 2.6.2 16S ribosomal RNA (rRNA) genetic analysis

Amplicons were adjusted for 16S RNA genetic analysis according to the manufacturer's protocol (16S Ribosomal RNA Gene Amplicons for the Illumina MiSeq System guidelines, Illumina, 2020). The V3–V4 region of the 16S rRNA gene of the extracted DNA was amplified using KAPA HiFi Hot Start Ready Mix (Roche) with the primer pair 341F (5′‐TCGTCGGCAGCGTCAGATGTGTATAAGAGACAGCCTACGGGNGGCWGCAG‐3′) and 806R primer (5′‐GTCTCGTGGGCTCGGAGATGTGTATAAGAGACAGGACTACHVGGGTATCTAATCC‐3′). Sequencing was performed for 600 cycles using Nextera XT Index Kit V2 (Illumina) as an adapter and the Miseq Reagent Kit v3 (Illumina) for pair ends. The FASTQ file obtained by Miseq measurement was analyzed using QIIME2 (2019.7), an integration analysis pipeline. Low‐quality sequence reads were filtered using the DADA2 package. The sequence errors and chimera sequences were filtered to estimate amplicon sequence variants (ASVs). The obtained ASVs were collated with the Human Oral Microbiome Database to assign bacterial species. The obtained sequence data have been deposited in DDBJ (DDBJ accession number: DRR385398–DRR385749).

#### 2.6.3 Bacterial counting

A quantitative polymerase chain reaction was performed to determine the total bacterial count. Amplification was performed using 2× Power SYBR Green PCR Master Mix (TAKARA BIO) according to the manufacturer's recommendations, using universal primers (Inubushi et al., 2010): forward primer (5′‐GTGSTGCAYGGYTGTCGTCA‐3′) and reverse primer (5′‐ACGTCRTCCMCACCTTCCTC‐3′). DNA was extracted from *Porphyromonas gingivalis* ATCC 33277 strain, adjusted for the bacterial count as the standard solution, and the bacterial count was calculated from the prepared calibration curve.

### 2.7 Statistical analysis

After excluding subjects (1) with missing data from Day 0, Day 1, and after 3 weeks; (2) not satisfying the eligibility criteria; (3) orally or intravenously taking substances (e.g., antibacterial drug, antibiotics) potentially affecting the oral microflora during the study; (4) requiring dental treatment; and (5) showing poor compliance with CPC spray (use rate of less than 80%), the remaining subjects were included in statistical analyses. Age was analyzed by the independent‐samples *t* test (*p* < .05), and the number of teeth, PPD, BOP, PCR, GI, and TCI were analyzed by the Mann–Whitney *U* test (*p* < .05). The changes in α‐diversity and total bacterial count were analyzed by the Friedman test (*p* < .05) using IBM SPSS Statistics ver. 27.0 (IBM Corp.). For the analysis of β‐diversity, principal component analysis (PCA) was performed using R language (ver. 3.6.1), with the FactoMineR and Factoextra packages. To confirm the difference between the CPC spray and control groups, a permutational multivariate analysis of variance (PERMANOVA) test (*p* < .05, permutations = 999) was performed using the vegan package. The bacterial characteristics in each group were determined by linear discriminant analysis (LDA) effect size (LEfSE), which was performed using the online program Huttenhower (https://huttenhower.sph.harvard.edu/galaxy/) (LDA > 2.0, *p* < .05).

## 3 RESULTS

### 3.1 Basic characteristics of study subjects

**Figure 1:**
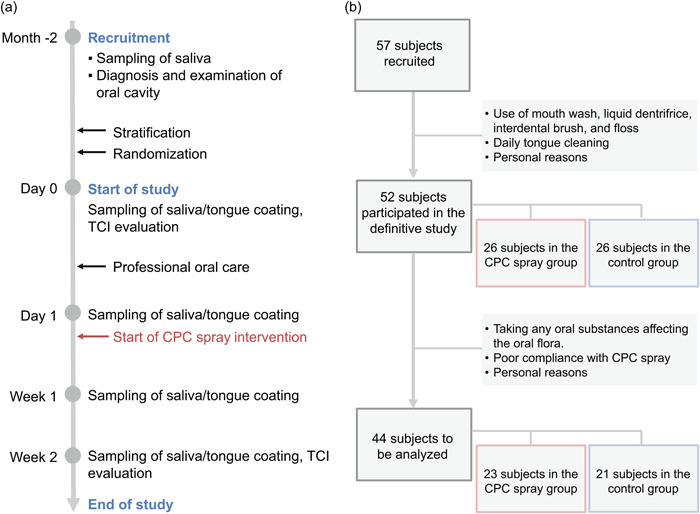
Study schedule and flowchart. (a) Study schedule. (b) A total of 57 subjects were recruited; after applying the exclusion criteria, 52 subjects were eligible to participate in this study; 26 subjects were allocated to each group, and 44 subjects were included in the final analysis. CPC, cetylpyridinium chloride; PMTC, professional mechanical tooth cleaning; SC, scaling; TCI, tongue coating index.

The study schedule and flowchart of recruitment are shown in Figure 1. Among the 57 recruited subjects, a total of five subjects with the habit of tongue cleaning and the habit of using a liquid preparation, and those with difficulty in participating in the study due to personal reasons were excluded, leaving 52 subjects eligible for participation in the definitive study. After allocation, there were 26 subjects in the CPC spray group and 26 subjects in the no‐use group (control group). After the start of the study, subjects were excluded owing to personal reasons, the use of a liquid preparation containing a microbicide, and less than 80% use of CPC spray, leaving a total of 23 subjects in the CPC spray group and 21 subjects in the control group.

The participants' background, age, sex, number of teeth, PPD, BOP, PCR, and GI confirmed at the time of recruitment are summarized in Table 1. There were no significant differences between the CPC spray and control groups in any of the baseline characteristics (*p* > .05).

**Table 1:** Basic characteristics of subjects in each group.

	All *n* = 44	CPC group *n* = 23	Control group *n* = 21	*p* Value
Gender(male/female)	15/29	9/14	6/15	.460
Age^a^	44 (8.3)	45 (8.0)	42 (8.6)	.270
Number of tooth^b^	28 [24–28]	28 [24–28]	28 [24–28]	.909
PPD (mm)^b^	2.1 [1.6–3.6]	2.2 [1.7–3.6]	2.0 [1.6–3.1]	.438
BOP (%)^b^	16.3 [0.6–48.2]	15.4 [1.8–48.2]	17.3 [0.6–39.9]	.906
PCR^b^	39.6 [1.8–79.5]	37.5 [9.8–70.5]	50.9 [1.8–79.5]	.622
GI^b^	0.67 [0.00–2.00]	0.67 [0.00–2.00]	0.67 [0.00–2.00]	.991

*Note*: Standard deviations are shown in parentheses, and variations are shown in square brackets.

Abbreviations: BOP, bleeding on probing; CPC, cetylpyridinium chloride; GI, gingival index; PCR, plaque control record; PPD, probing pocket depth.

^a^ Student's *t* test for independent samples.

^b^ Mann–Whitney *U* test

### 3.2 β‐Diversity by PCA

**Figure 2:**
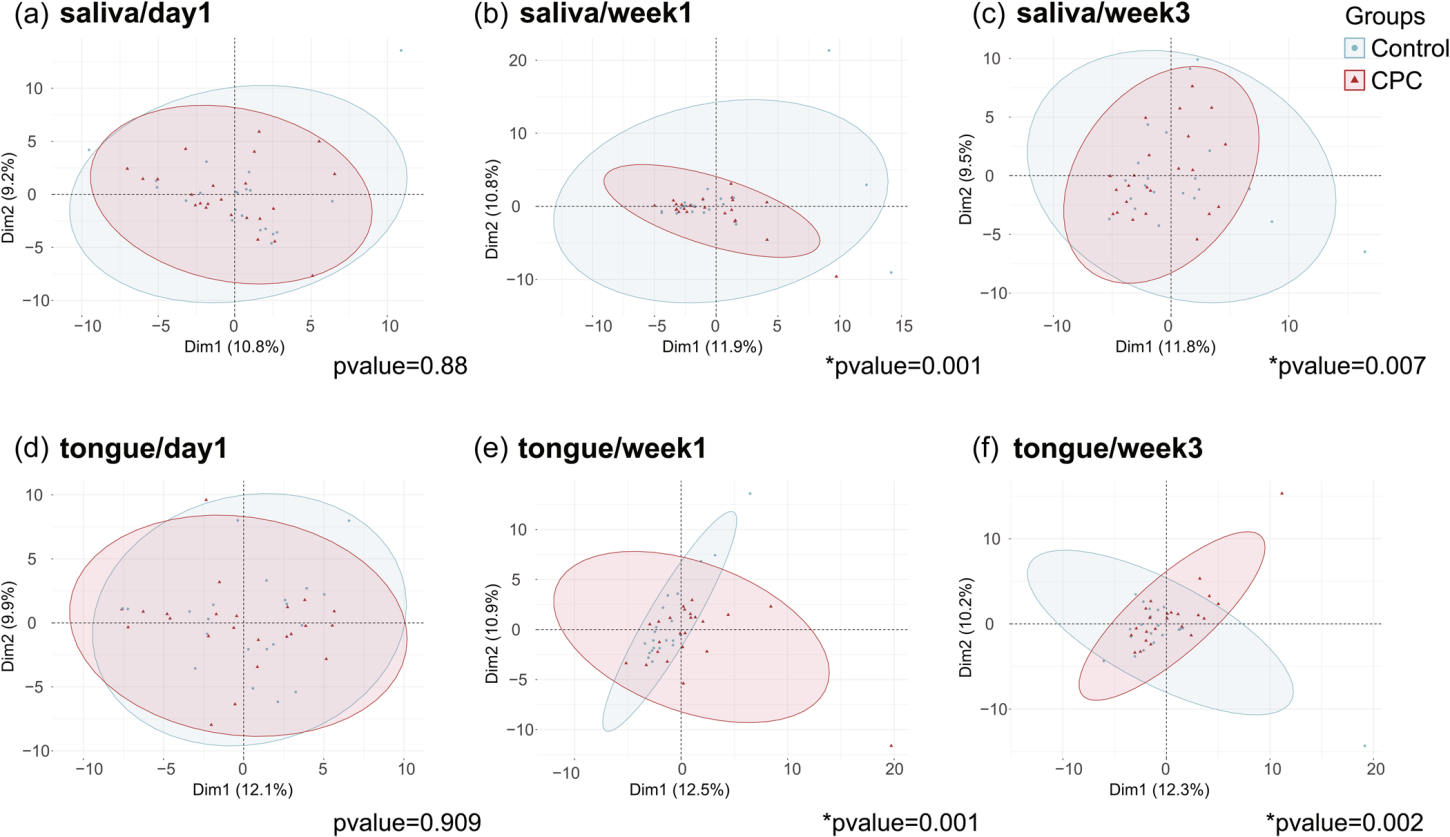
Change in β‐diversity based on principal component analysis. (a) Saliva/Day 1, (B) saliva/Week 1, (c) saliva/Week 3, (d) tongue/Day 1, (e) tongue/Week 1, (f) tongue/Week 3. Blue circles indicate the control group and red triangles indicate the cetylpyridinium chloride (CPC) spray group. *p < .05, permutational multivariate analysis of variance test.

The results of the β‐diversity analysis of the saliva and tongue coating samples are shown in Figure 2. For both the saliva and tongue coating samples, there was no significant difference in the diversity of the oral microflora between the CPC spray group and the control group on Day 1 (saliva *p* = .88, tongue *p* = .909); however, the β‐diversity of the microflora was significantly different between the CPC spray group and the control Group at 1 and 3 weeks after the intervention (PERMANOVA, saliva/Week 1 *p* = .001, saliva/Week 3 *p* = .007, tongue/Week 1 *p* = .001, tongue/Week 3 *p* = .002).

### 3.3 Changes in the bacterial flora composition and count in the saliva and tongue coating

**Figure 3:**
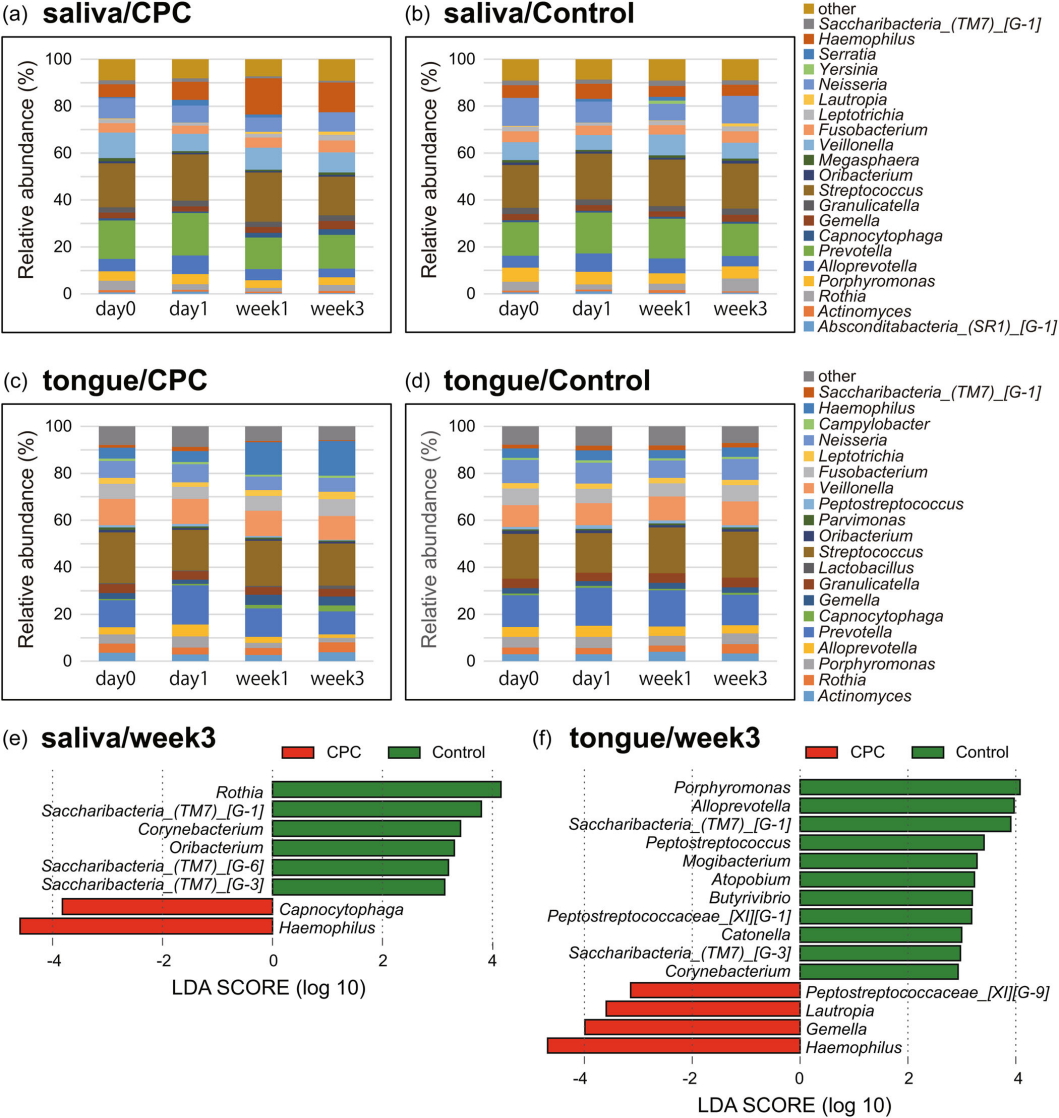
Changes in oral microflora at each sampling time. Levels of bacterial genus with an occupancy of 1% or more are shown, and those of less than 1% are described as “other.” (a) saliva/CPC spray group. (b) Saliva/control group. (c) Tongue/CPC spray group. (d) Tongue/control group. LEfSe analysis was performed 3 weeks after the intervention with CPC spray. (e) Saliva/3 weeks and (f) tongue/3 weeks, the CPC spray group is shown in red and the control group is shown in green. CPC, cetylpyridinium chloride; LDA, linear discriminant analysis; LEfSe, LDA effect size.

The change in oral microflora at the genus level for each sampling time of saliva and tongue coating samples is shown as a bar chart in Figure 3. Bacterial genus with an occupancy of 1% or more were extracted, and bacterial genus with an occupancy of less than 1% were described as “other.” On Day 1, an increase in genus *Prevotella* was detected in both groups for the saliva and tongue coating samples in comparison with that at Day 0. One week after the intervention with CPC spray, for both the saliva and tongue coating samples, an increase in genes *Haemophilus* was observed in the CPC spray group, and the change persisted for 3 weeks. In the control group, there was no significant change in microflora composition compared to that in the CPC spray group (Figure 3). Moreover, the characteristic bacterial genus in the CPC spray and control groups after 3 weeks were extracted by LEfSe analysis (Figure 3). For both the saliva and tongue coating samples, genus *Haemophilus* was characterized in the CPC spray group, whereas genus *Saccharibacteria* was characterized in the control group. Moreover, in the saliva samples, genus *Capnocytophaga* was characterized in the CPC spray group, whereas genus *Rothia*, *Saccharibacteria*_ (TM7)_[G‐1], *Corynebacterium*, *Oribacterium*, *Saccharibacteria*_(TM7)_[G‐6], and *Saccharibacteria*_(TM7)_[G‐3] were characterized in the control group. In the tongue coating samples, more characteristic bacterial genus were detected in each group than found in the saliva samples: genus *Gemella*, *Lautropia*, and Peptostreptococcaceae_[XI][G‐9] were detected in the CPC spray group, and a total of 11 bacterial genera, including *Porphyromonas*, *Alloprevotella*, *Saccharibacteria*_(TM7)_[G‐1], *Peptostreptococcus*, *Mogibacterium*, *Atopobium*, *Butyrivibrio*, Peptostreptococcaceae_[XI][G‐1], *Catonella*, *Saccharibacteria*_(TM7)_[G‐3], and *Corynebacterium* were detected in the control group (Figure 3).

Changes in the total bacterial count in the saliva and tongue coating samples are shown in Figure S1. In both the CPC spray group and the control group, a significant difference was partially confirmed in the saliva and tongue coating samples. In all samples/groups, the total bacterial count tended to increase on day 1 compared to that at Day 0.

### 3.4 Change in the TCI

**Figure 4:**
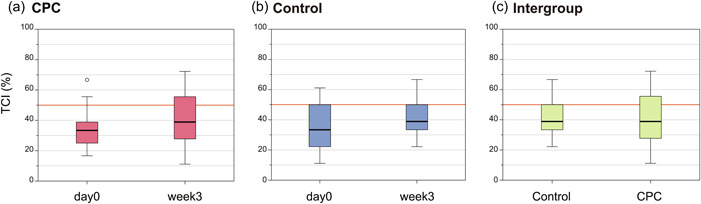
Change in the tongue coating index (TCI). (a) Change over time in the cetylpyridinium chloride (CPC) spray group and (b) control group. (c) Intergroup change at 3 weeks between the CPC spray group and the control group. The orange line indicates 50%, the judgment criterion for a poor oral hygienic condition. The Mann–Whitney *U* test was performed and no significant difference was observed (*p* > .05).

The change in the TCI was determined on Day 0 (start of the study) and 3 weeks after the intervention with CPC spray (Figure 4). No significant intragroup change was observed in either the CPC spray group or the control group, although the median TCI value tended to increase. No significant intergroup difference in the TCI was observed after 3 weeks between the CPC spray and control groups.

## 4 DISCUSSION

In this study, a change in the oral bacterial flora of the saliva/tongue coating was observed after intervention with CPC spray for 3 weeks after professional oral care. To allocate the condition of oral flora evenly to the CPC spray group and the control group, the subjects were stratified according to the high and low values of the Shannon index, an index of α‐diversity, of the salivary flora collected at the time of recruitment. After randomization, there were no significant differences in the clinical background between groups.

There was no significant difference between the groups in the β‐diversity analysis on Day 1. This is due to the lack of difference in intraoral clinical indices between the CPC spray group and the control group, and professional oral care was performed in all subjects to remove the supragingival plaque before starting the study. However, after 1 and 3 weeks, the diversity of the microflora significantly changed between the CPC spray group and the control group, which is considered to reflect the effect of the intervention with the CPC spray.

Regarding the specific changes in microflora according to LEfSe analysis, genus *Haemophilus* markedly increased in the saliva and tongue coating samples after 1 and 3 weeks in the CPC spray group compared to the control group. Barbagallo et al. (2021) reported that genus *Haemophilus* represents part of the normal core bacterial species. Al‐Kamel et al. (2019) showed that genus *Haemophilus* was identified in subjects with healthy gingiva, and Belstrøm et al. (2017) reported that the proportion of this species is high in the saliva sample of individuals with minimal experience of caries. Among species of the genus *Haemophilus*, *H. influenzae* is a causative bacterium of pneumonia and *H. parainfluenzae* is considered to survive in the oral cavity of healthy individuals. Confirming the occupancy of genus *Haemophilus* at the species level 1 and 3 weeks after intervention with CPC spray, the occupancy of *H. parainfluenza*e was the highest (Table S1). Perera et al. (2021) reported that *H. parainfluenzae* is commonly present in saliva samples of people with minimal experience of caries, and Belstrøm et al. (2017) confirmed that *H. parainfluenzae* is a bacterial species constituting the supragingival plaque of healthy people. Diao et al. (2021) reported that low occupancy of this bacterial species is a biomarker of periodontal disease. Moreover, in a study by Teng et al. (2016), genus *Haemophilus* increased in the group using CPC mouth rinse, and a reproducible increase in genus *Haemophilus* was observed using a CPC preparation. However, the detailed mechanism of the increase in genus *Haemophilus* after the use of a CPC preparation has not been clarified.

Genus *Lautropia* was also extracted in the LEfSe analysis of the CPC spray group. A previous study reported that the abundance ratio of genus *Lautropia* in the subgingival plaque of healthy people was higher than that in subjects with gingivitis and peri‐implantitis (Barbagallo et al., 2021), and was also high in people with a decayed, missing, and filled teeth index of 0, an empirical value for caries (Xiao et al., 2016). Moreover, Schwartz et al. (2021) and Ikeda et al. (2020) showed that the subclass *Lautropia mirabilis* is frequently identified in subjects with a healthy gingiva (Al‐Kamel et al., 2019). Moreover, the genus *Saccharibacteria*, which was detected as a characteristic species in the control group in the present study, was previously reported to be enriched at the site of gingivitis in comparison with the healthy site (Nibali et al., 2020) and was also reported as bacteria related to caries (da Costa Rosa et al., 2021). However, genus *Saccharibacteria* was only cultured for the first time in 2015. Chipashvili et al. (2021) suggested that this genus may inhibit inflammation induced by host bacteria and reduce the function involved in pathogenicity, although the details remain unclear and warrant further investigation.

No significant change in the TCI was noted after the intervention with CPC spray on day 0 and after 3 weeks. The TCI is one of the indices used to diagnose oral hypofunction in the elderly population in Japan (Minakuchi et al., 2018). The TCI is also used as a general index to evaluate oral hygiene conditions; a TCI of 50% is considered to indicate an unclean oral cavity (Minakuchi et al., 2018). In this study, the median TCI in both groups was below 50%, indicating that this group had relatively good oral hygiene; therefore, it was considered that the change in TCI was small. Although the intervention with CPC spray changed the lingual flora, a decrease in TCI was not confirmed, suggesting that it may be necessary to combine physical cleaning with a tongue brush for appropriate tongue care.

The total bacterial count increased from Day 0 to 1 despite the intervention of professional oral care, which is considered to be due to the effects of the timing of sampling on diurnal variation. Since the subjects visited the dental clinic for sampling on Day 0 and after 3 weeks, there was variation in sampling time among individuals. However, the sample after waking up was collected at the home of the subjects on Day 1 and after 1 week. Since the time period directly after waking up is when the bacterial count is the highest in the day (Nolte, 1982), this was considered to reflect the increase observed from Day 0 to 1 in this study (Figure S1).

The limitations of this study are as follows. First, the identification of bacterial species with low occupancy is poor in 16S rRNA genetic analysis. Second, since the sequences of related species are similar in the V3–V4 region of the 16S rRNA gene, the accuracy of allocation at the species level is insufficient. Third, it is possible that the timing of sample collection was not aligned, which may have affected the bacterial count results. The sampling time of four times was not aligned within an individual and it differed among participants, especially on Day 0 and Week 3. To our knowledge, this is the first study of microflora in the saliva and tongue coating in a clinical evaluation after intervention with CPC spray; however, further study is necessary to explore the detailed changes in oral microflora induced by a CPC preparation. In conclusion, the microflora in the saliva/tongue coating changed with the use of CPC spray, with a particular change in genus *Haemophilus*. However, no change in the TCI was observed, it may be necessary to combine physical cleaning with a tongue brush.

## AUTHOR CONTRIBUTIONS

Haruko Tobata, Hirohisa Suido, and Toru Eguchi conceived the idea of the study. Ai Fujimoto, Kana Fujii, Haruko Tobata, and Hirohisa Suido developed the statistical analysis plan and Ai Fujimoto conducted statistical analyses. Hidenori Suzuki, Naoko Miyake, Hisae Fukuike, and Kana Fujii contributed to collect the clinical data and interpretation of the results. Ai Fujimoto, Toru Eguchi, and Haruko Tobata contributed to the interpretation of the bacteriological results. Ai Fujimoto and Kana Fujii drafted the original manuscript. Haruko Tobata supervised the conduct of this study. All authors reviewed the manuscript draft and revised it critically on intellectual content. All authors approved the final version of the manuscript to be published.

## CONFLICTS OF INTEREST STATEMENT

Ai Fujimoto, Kana Fujii, Toru Eguchi, and Haruko Tobata are employees of Sunstar Inc., and Hisae Fukuike, Naoko Miyake, and Hidenori Suzuki are employees of the Sunstar Foundation. The remaining author declares no conflict of interest.

## ETHICS STATEMENT

This study was reviewed by the Ethics Committee of the Japanese Society for Oral Health (approval no.: 2020‐1), was registered with the University Hospital Medical Information Network (UMIN; UMIN000041140), and was conducted according to the ethical principles of the Declaration of Helsinki. Informed consent was obtained from all subjects before participation in the study.

## Supporting information

Supporting information.

Supporting information.

## Data Availability

The 16S ribosomal RNA sequencing data is available. They have been deposited in DDBJ (DDBJ accession number: DRR385398–DRR385749).
