# Supplementary material for: Changes in oral microflora following 0.3% cetylpyridinium chloride‐containing mouth spray intervention in adult volunteers after professional oral care: Randomized clinical study
Source: Clin Exp Dent Res. 2023 Dec 2;9(6):1034–43. doi: 10.1002/cre2.810 (PMC10728501; doi:10.1002/cre2.810)
Supplement: Supplementary file 2 — Supporting information. [file CRE2-9-1034-s002.docx]

**Supplementary Information**

**Changes in Oral Microflora Following 0.3% Cetylpyridinium Chloride-Containing Mouth Spray Intervention in Adult Volunteers after Professional Oral Care: Randomized Clinical Study**

**Authors:** Ai Fujimoto, Kana Fujii, Hirohisa Suido, Hisae Fukuike, Naoko Miyake, Hidenori Suzuki, Toru Eguchi, and Haruko Tobata

**Corresponding author:** Ai Fujimoto, Research and Development, Sunstar Inc., Osaka, Japan

**E-mail:** [ai.fujimoto@jp.sunstar.com](mailto:ai.fujimoto@jp.sunstar.com)

**Supplementary Table S1**. Allocation of genus *Haemophilus* to the species level (xlsx file).

The number of reads in the saliva and tongue coating samples at the time of sampling after one and three weeks at the species level is shown.


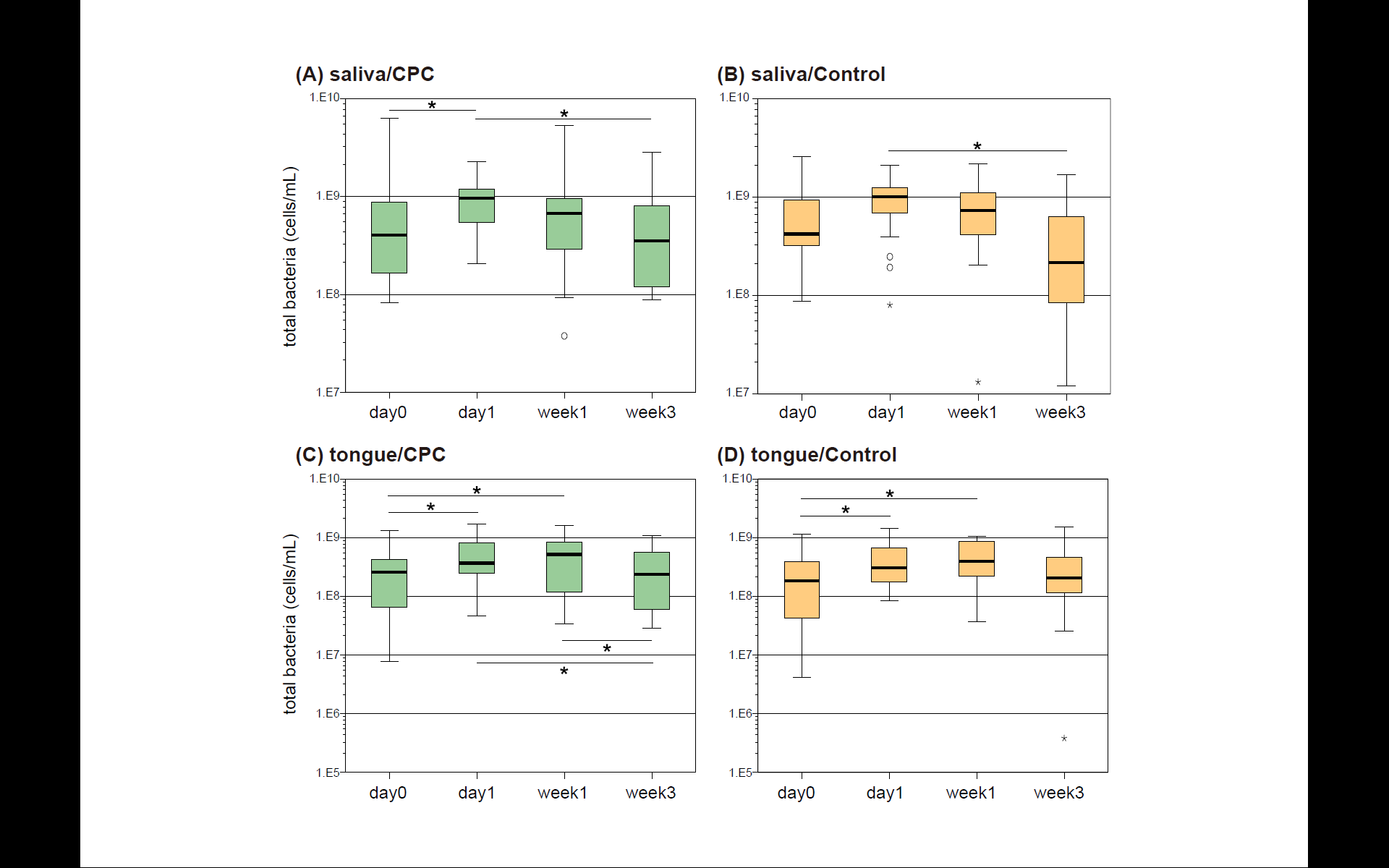


**Supplementary Figure S1. Measurement of total bacterial count** **at each sampling time.** (A) Saliva/CPC spray group, (B) saliva/control group, (C) tongue/CPC spray group, (D) tongue/control group. **p* < 0.05, Friedman test.
